# Insight into the early steps of root hair formation revealed by the *procuste1 *cellulose synthase mutant of *Arabidopsis thaliana*

**DOI:** 10.1186/1471-2229-8-57

**Published:** 2008-05-16

**Authors:** Sunil K Singh, Urs Fischer, Manoj Singh, Markus Grebe, Alan Marchant

**Affiliations:** 1 Department of Forest Genetics and Plant Physiology, SLU, 901 83 Umeå, Sweden; 2 Institute of Biology II, University of Freiburg, Schänzlestrasse 1, 79104 Freiburg, Germany; 3 School of Biological Sciences, University of Southampton, Boldrewood Campus, Southampton. SO16 7PX, UK; 4 Department of Plant Physiology, Umeå University, 90187 Umeå, Sweden; 5 Georg-August University, Göttingen, Germany

## Abstract

**Background:**

Formation of plant root hairs originating from epidermal cells involves selection of a polar initiation site and production of an initial hair bulge which requires local cell wall loosening. In Arabidopsis the polar initiation site is located towards the basal end of epidermal cells. However little is currently understood about the mechanism for the selection of the hair initiation site or the mechanism by which localised hair outgrowth is achieved. The Arabidopsis *procuste1 *(*prc1-1*) cellulose synthase mutant was studied in order to investigate the role of the cell wall loosening during the early stages of hair formation.

**Results:**

The *prc1-1 *mutant exhibits uncontrolled, preferential bulging of trichoblast cells coupled with mislocalised hair positioning. Combining the *prc1-1 *mutant with *root hair defective6-1 *(*rhd6-1*), which on its own is almost completely devoid of root hairs results in a significant restoration of root hair formation. The *pEXPANSIN7::GFP *(*pEXP7::GFP*) marker which is specifically expressed in trichoblast cell files of wild-type roots, is absent in the *rhd6-1 *mutant. However, *pEXP7::GFP *expression in the *rhd6-1/prc1-1 *double mutant is restored in a subset of epidermal cells which have either formed a root hair or exhibit a bulged phenotype consistent with a function for EXP7 during the early stages of hair formation.

**Conclusion:**

These results show that *RHD6 *acts upstream of the normal cell wall loosening event which involves *EXP7 *expression and that in the absence of a functional RHD6 the loosening and accompanying *EXP7 *expression is blocked. In the *prc1-1 *mutant background, the requirement for RHD6 during hair initiation is reduced which may result from a weaker cell wall structure mimicking the cell wall loosening events during hair formation.

## Background

Root hairs are slender projections originating from epidermal cells that function in nutrient and water uptake as well as in anchoring the root in the soil [1]. In wild-type Arabidopsis, root hairs are formed by epidermal cells termed trichoblasts which overlie the boundary between two cortical cells [2]. The formation of a root hair can be divided into two distinct stages, namely initiation and outgrowth [3]. The first detectable marker of root hair initiation is the appearance of a Rop GTPase which is localised towards the basal end of trichoblasts prior to any visible bulge formation [4,5]. The first visible sign of root hair initiation is characterized by the formation of a bulge which in Arabidopsis is typically located towards the basal end of the epidermal cell [6,7]. In order for the bulge to form, the cell wall must undergo loosening and it is thought that alkalinisation of the cytoplasm, acidification of the cell wall [8], expansin (EXP) and xyloglucan endotransglycosylase (XET) activity [9] all contribute to this step. XETs act by breaking and reforming the glycosidic bonds of xyloglucan which cross links cellulose microfibrils whereas the expansins mediate cell wall loosening without undergoing breakage of the major structural components of the cell wall. XET activity has been demonstrated to be localized to the site of root hair bulge formation [9], suggesting a specific role in hair formation. Two of the Arabidopsis expansin genes (*AtEXP7 *and *AtEXP18*) are expressed in trichoblast but not atrichoblast cells [10], indicating that they also play a role in loosening of the cell wall to promote hair initiation and outgrowth. The role of expansins in root hair formation is further substantiated by the finding that they accumulate at the site of bulge formation in maize roots [11]. Additionally, in barley the *HvEXPB1 *expansin gene expression is absent in the root hairless *bald root barley *mutant but is normal in 2 mutants which form short root hairs. This suggests that the HvEXPB1 is required for the initiation of root hairs [12].

Cellulose is a major structural component of cell walls comprising chains of β-1,4-linked glucosyl residues which are assembled into microfibrils. The arrangement of the microfibrils in the cell wall influences the manner in which cells expand. A number of mutants have been described in Arabidopsis which exhibit abnormal cell expansion and several of these are affected in cellulose biosynthesis. For example, abnormal radial swelling is observed in the *rsw1 *(*CESA1*; [13]), *rsw2 *(*KORRIGAN*; [14,15]); *rsw3 *(glucosidase II; [16]) and *rsw10 *(ribose-5-phosphate isomerase; [17]) mutants. The *rsw10 *mutant exhibits ballooning of root trichoblast cells that is thought to arise from the cellulose deficiency in the root. Interestingly, the expression of *RSW10 *is not limited to the trichoblast cell files providing a possible link between root hair formation and abnormal expansion in *rsw10*. The *root epidermal bulger *(*reb1/rhd1*) mutant of Arabidopsis also exhibits abnormal expansion of trichoblast cells [18,19]. *REB1 *encodes an isoform of UDP-D-glucose 4-epimerase which functions in forming UDP-D-galactose. The *reb1 *mutant lacks galactosylated xyloglucan and arabinosylated (1→6)-β-D-galactan [20]. Interestingly, the *reb1 *mutant shows a loss of the JIM14 and LM2 arabinogalactan epitope in trichoblasts while it remains in atrichoblasts [19] implying that trichoblast arabinogalactan proteins (AGPs) are required for normal anisotropic expansion.

In this study we have made use of the *procuste1 *(*prc1-1*) cellulose deficient mutant of Arabidopsis to probe the influence of cell wall structure in modulating root hair formation. The Arabidopsis *prc1-1 *is mutated in the cellulose synthase *CESA6 *gene resulting in a reduction in the cellulose content of the cell walls [21]. Mutant *prc1-1 *seedlings exhibit bulging of the hair forming trichoblast cells of the root and a reduction in primary root elongation [21,22]. Our studies show that *prc1-1 *is able to partially bypass the defect in hair formation of the *rhd6-1 *root hairless mutant, demonstrating that the cell wall structure is an important factor during hair morphogenesis.

## Results

### Root epidermal bulging in *prc1-1 *is predominantly associated with trichoblast cells

**Figure 1:**
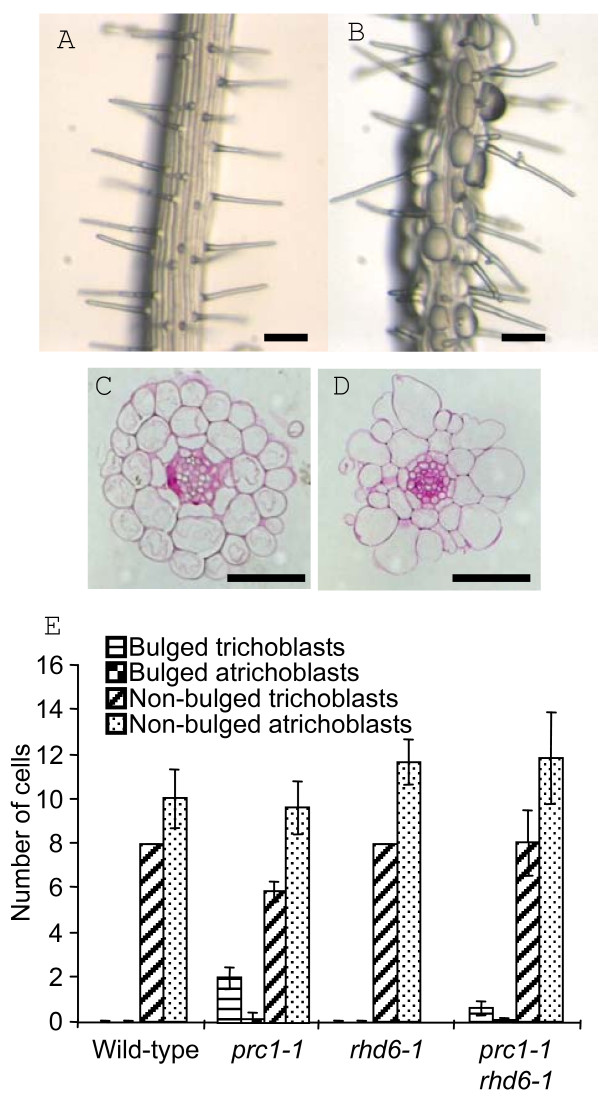
***prc1-1 *roots exhibit epidermal bulging predominantly in trichoblast cells**. Wild-type (A) and *prc1-1 *roots (B) grown on MS agar for 5 days. Radial sections of wild-type (C) and *prc1 *roots (D). (E) Quantification of the number of bulged and non-bulged epidermal cells in the trichoblast or atrichoblast positions of wild-type, *prc1-1, rhd6-1 *and *prc1-1/rhd6-1 *mutants. Error bars show sd. Bar = 100 μm A, B; 50 μm, C, D.

Despite previous descriptions of the *prc1-1 *phenotype, detailed analysis of the root defects and in particular, the phenomenon of the bulging epidermal cells has not been performed. Examination of *prc1-1 *roots shows that the bulged cells are predominantly arranged in apical-basal oriented files flanked by files of non bulged cells (Fig. 1B). This indicates that the bulging phenomenon in *prc1-1 *roots may not be a random event but linked in some way to the positional properties of particular cells. To investigate this further, radial sections were made of resin embedded roots of *prc1-1 *and wild-type seedlings and each bulged or non-bulged cell scored as to whether it was in a trichoblast (hair-cell) or atrichoblast (non-hair cell) position. The bulged cells were predominantly though not exclusively located in the trichoblast cells of the *prc1-1 *roots (Fig. 1D, E). No evidence of epidermal cell bulging was found in wild-type root tissues (Fig. 1A, C). This shows that the bulging of epidermal cells is associated with their radial position with respect to the underlying cortical cells and that it may be linked with the process of hair initiation and/or outgrowth.

Although bulged epidermal cells often formed a root hair it was evident that hairs were also formed by non-bulged cells and some bulged cells failed to form a root hair. From a sample size of 500 cells from 50 independent primary roots which did not include unbulged cells lacking a root hair, the majority (65%) were bulged and had formed a hair. Bulged cells which had not formed a hair (17%) and cells which were not bulged but had formed a hair (18%) were evident less frequently but in similar proportions. Although the developmental events leading to hair formation in *prc1-1 *normally result in a bulged cell phenotype it is possible for hairs to be formed by cells which are not bulged.

### Mutant *prc1-1 *roots have reduced epidermal cell length and increased root hair density

**Figure 2:**
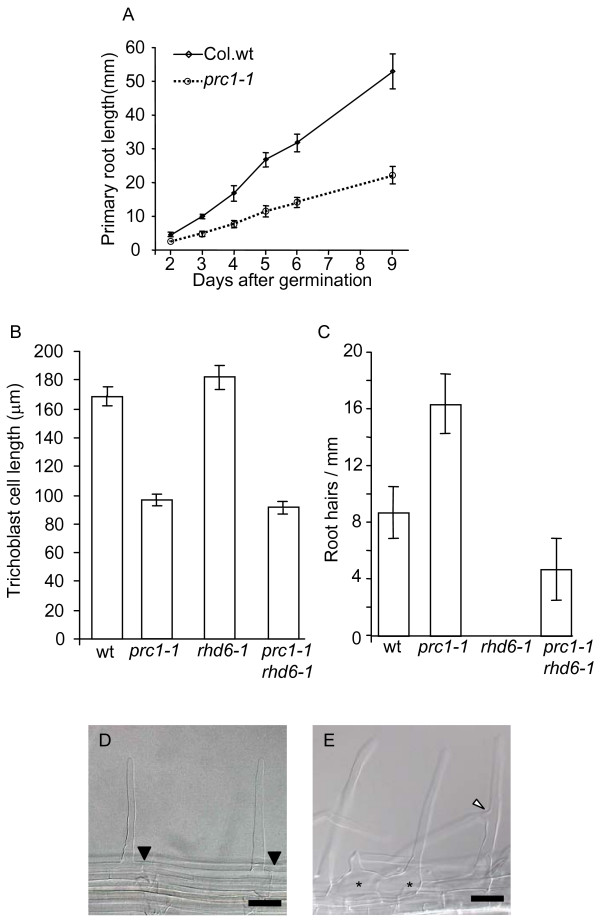
**Primary root growth and hair development is affected in the *prc1-1 *mutant**. (A) Root elongation of wild-type and *prc1-1 *between 2 and 9 days after germination. (B) Trichoblast cell lengths in wild-type, *prc1-1*, *rhd6-1 *and *prc1-1/rhd6-1 *primary roots (n = 600). (C) Number of root hairs per mm in trichoblast cell files of 7 day old wild-type, *prc1-1*, *rhd6-1 *and *prc1-1/rhd6-1 *seedlings. (D) Wild-type root showing single root hairs which have originated from the basal end of epidermal cells. Filled triangles mark the position of the basal walls of the hair forming epidermal cells. (E) *prc1-1 *root showing 2 hairs originating from a single epidermal cell (*) and a branched root hair where the branch point is highlighted by an open triangle. Error bars show SEM (B) or SD (C). Bar = 50 μm.

The *prc1-1 *roots are shorter than the wild-type (Fig. 2A) and have a more hairy appearance. This may be a consequence of *prc1-1 *having a reduced epidermal cell length or alternatively the mutant may form more root hairs compared to wild-type. To test these possibilities wild-type and *prc1-1 *roots were cleared and the trichoblast cell lengths measured in differentiated root hair cells. Mutant *prc1-1 *trichoblast cells were 43% reduced in length compared to the wild-type (Fig. 2B). Thus, the shorter *prc1-1 *root length is primarily due to reduced cell elongation rather than decreased cell division. To further determine the basis for the apparent increased hair density in *prc1-1 *roots, the numbers of hairs per mm were counted in separate files of trichoblast cells. This shows that *prc1-1 *root hair density is almost double that of the wild-type (Fig. 2C). Although the majority of *prc1-1 *trichoblast cells formed a single hair, 4% of epidermal cells were observed having two root hairs (n = 358 cells) (Fig. 2E). In contrast, wild-type trichoblast cells only ever formed single hairs (n = 236 cells) (Fig. 2D). It was also notable that *prc1-1 *had a significant degree of branching of the root hairs (Fig. 2E). Thus the appearance of a higher density of root hairs in *prc1-1 *is largely due to the decrease in epidermal cell length with a minor contribution from the double hair and hair branching phenotypes.

### The polarity of Rop localisation and root hair positioning is variable in *prc1-1*

**Figure 3:**
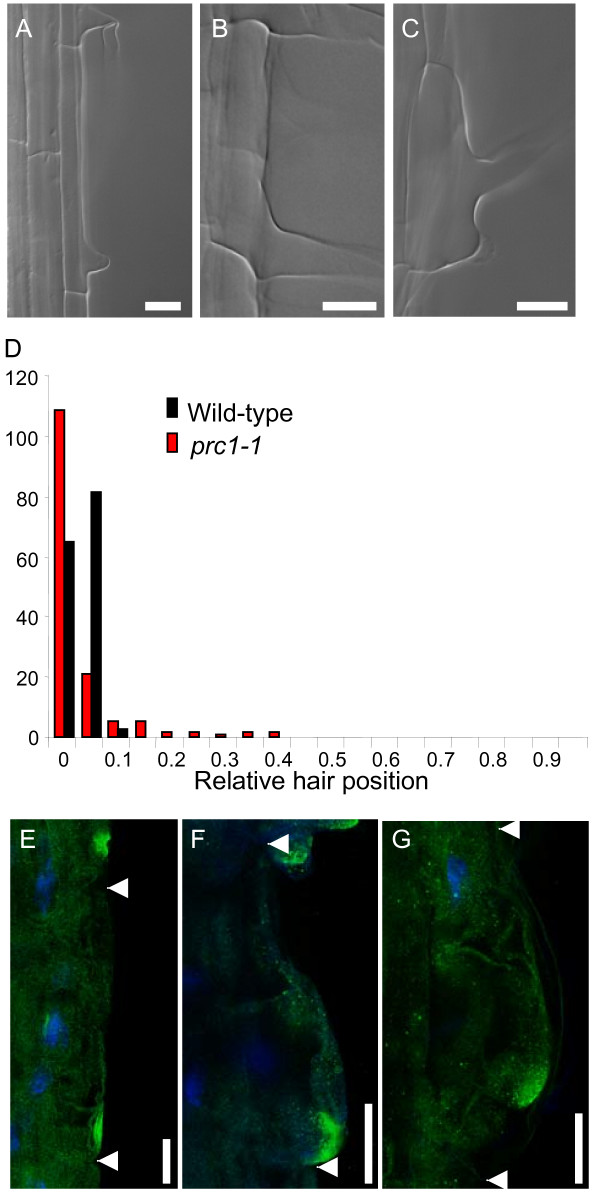
**Localisation of root hair formation is altered in the *prc1-1 *mutant**. The positions of root hairs relative to the basal cell wall were measured for wild-type and *prc1-1 *seedlings. (A) Wild-type showing typical root hair emergence towards the basal end of the trichoblast. (B) *pcr1-1 *showing a basally hyperpolarized root hair. (C) *prc1-1 *showing an apically shifted root hair. (D) Frequency distribution of relative root hair position for wild-type and *prc1-1*. The Rop protein was localized in wild-type and *prc1-1 *roots using a specific anti-Rop antibody and DAPI staining was used to highlight the position of the nucleus. The Rop signal is shown in green and DAPI in blue (E) wild-type (F) basally hyperpolarised Rop signal in *prc1-1 *(G) apically shifted Rop signal in *prc1-1 *trichoblast. Arrowheads indicate the location of lateral cell-cell interfaces. Bar = 50 μm (A, B, C) 20 μm (E, F, G).

In wild-type roots, hairs normally initiate and emerge towards the basal end of trichoblast cells [6,7,23]. The site of future hair formation towards the basal end of trichoblasts is marked by the Rop GTPase prior to any visible bulge formation and thus represents a very early event in root hair initiation [4,5]. The formation of double hairs by a proportion of *prc1-1 *epidermal cells (Fig. 2E) indicates that the control of the site of hair initiation is affected. To test this, the position of hair formation relative to the basal ends of the epidermal cells was measured for wild-type and *prc1-1 *mutant seedlings. The distribution of hair positions in *prc1-1 *was determined to be significantly different from the wild-type using either a Fisher Exact test (please see Availability & requirements section below) using 2 × 5 tables (p = 0.0) or Mann-Whitney rank sum test (please see Availability & requirements section below) (p < 0.05), (Fig. 3D). There was predominantly a shift by *prc1-1 *root hairs towards a more basal position compared to wild-type (Fig. 3A, B). In addition to the basally shifted hairs in *prc1-1 *there were also a smaller proportion of the hairs that showed a shift towards more apical positions (Fig. 3C).

Immunolocalisation of the Rop GTPase was performed on wild-type and *prc1-1 *roots to determine whether there is also a shift in the polarity of this early root hair positional marker in the mutant [24]. In wild-type Arabidopsis roots the Rop signal is localised towards the basal ends of trichoblast cells consistent with the subsequent position of the root hair (Fig. 3A, E). In *prc1-1 *Rop signal is also found in some cells to be localized towards the basal region of the trichoblasts but it was noted that the signal was less discrete than in the wild-type and was often localised to a more extreme basal position (Fig. 3B, F). However in a proportion of epidermal cells there was a clear shift of the Rop signal to a more apical location (Fig. 3C, G) consistent with the observed shift in hair positioning in a subset of the *prc1-1 *epidermal cells (Fig. 3D).

### The expression pattern indicated by *PRC1 *promoter activity cannot account for predominance of bulging in trichoblast cells compared to atrichoblasts

**Figure 4:**
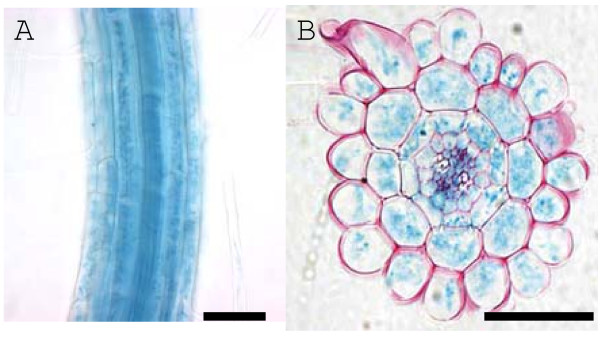
***PRC1 *promoter driven GUS expression is detected in all cells of the root**. Whole mount (A) and radial section (B) of a 7 day old p*PRC1*::*uidA *primary root stained for GUS activity. Bar = 50 μm.

The preferential epidermal bulging in *prc1-1 *trichoblasts compared to atrichoblasts (Fig. 1D, E) suggests there is a link with a trichoblast-specific characteristic or function. Alternatively, *PRC1 *expression may be limited to the trichoblast cell files accounting for a specific defect in this subset of epidermal cells. To test these possibilities a p*PRC1*::*uidA *line was stained for GUS activity which revealed that expression is apparent in all epidermal cells of the root as well as the underlying cortical, endodermal and stele cells (Fig. 4A, B). Thus, the predominance of epidermal bulging in trichoblasts cannot be accounted for by differential *PRC1 *promoter activity in trichoblasts compared to atrichoblasts. The possibility that the activity of the PRC1 protein or that of the primary cell wall cellulose synthase complex differs between the two epidermal cell types cannot be discounted.

### The *prc1-1 *mutation can partially rescue root hair formation in the *rhd6-1 *mutant

**Figure 5:**
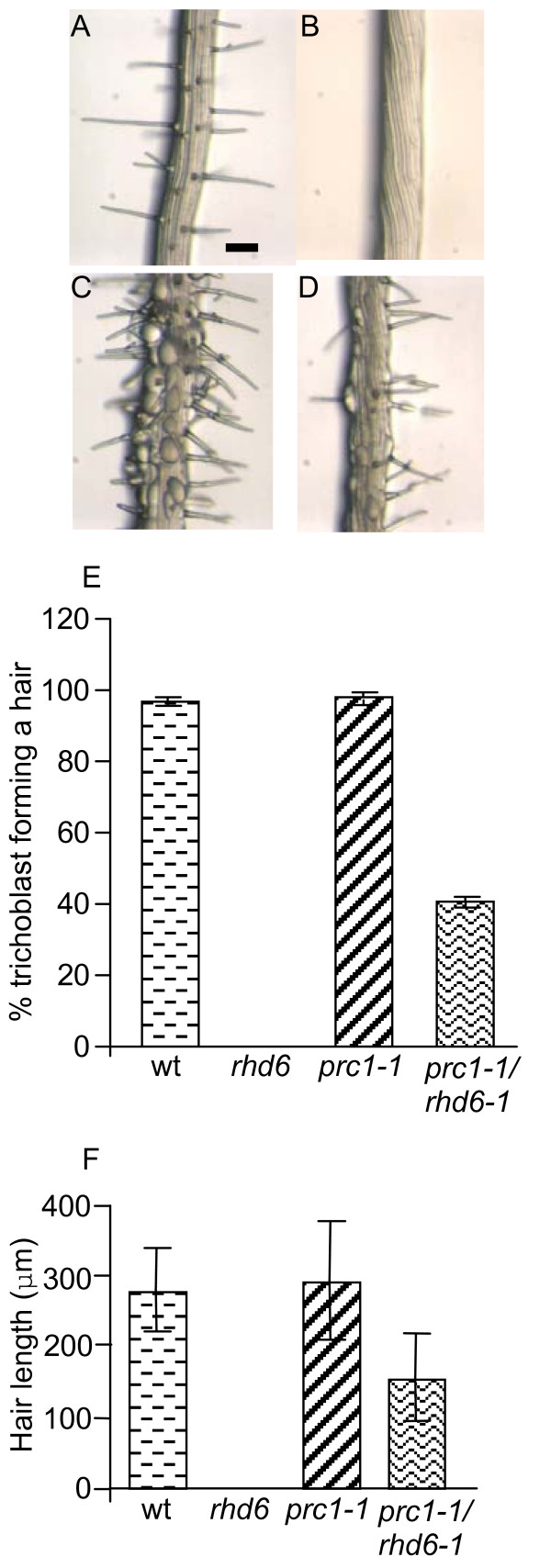
**Root hair development in *rhd6-1 *is partially restored in the *prc1-1 *mutant background**. Primary root tissues of wild-type (A), *rhd6-1 *(B), *prc1-1 *(C) and *rhd6-1/prc1-1 *(D) seedlings which have been grown on MS agar for 5 days. (E) The percentage of trichoblast cells showing root hair formation in wild-type, *rhd6-1*, *prc1-1 *and *prc1-1/rhd6-1 *(n = 600 cells). (F) Root hair lengths of wild-type, *rhd6-1*, *prc1-1 *and *prc1-1/rhd6-1 *(n = 100). Error bars show SEM (E) or SD (F). Bar = 100 μm.

If trichoblast cell bulging is linked to hair formation then it can be hypothesized that the bulging will be reduced or abolished when *prc1-1 *is in the *rhd6-1 *mutant background that is largely devoid of hairs [6]. To test this theory, double mutants were made between *prc1-1 *and *rhd6-1 *and their roots examined for the appearance of bulged root epidermal cells. The occurrence of bulged cells and the size of the bulges that formed were significantly reduced in the *prc1-1/rhd6-1 *double mutant (Fig. 5D) compared to *prc1-1 *(Fig. 1E, 5C). It was striking to note that there were a significant number of root hairs formed by the *prc1-1/rhd6-1 *double mutant (Fig. 2C, 5D) although the proportion of trichoblasts forming a root hair was less than half that of either the wild-type or *prc1-1 *single mutant roots (Fig. 5E). The morphology of the hairs that formed in the *rhd6-1/prc1-1 *double mutant appeared similar to those of the wild-type though they were reduced in length (Fig. 5F). In contrast *rhd6-1 *roots did not form any hairs under our growth conditions when grown on MS agar (Fig. 2C, 5B). This demonstrates that the *prc1-1 *defect is able to partially bypass the requirement for RHD6 in root hair formation.

### EXP7 expression in *rhd6-1 *root epidermal cells is partially restored in *prc1-1*

**Figure 6:**
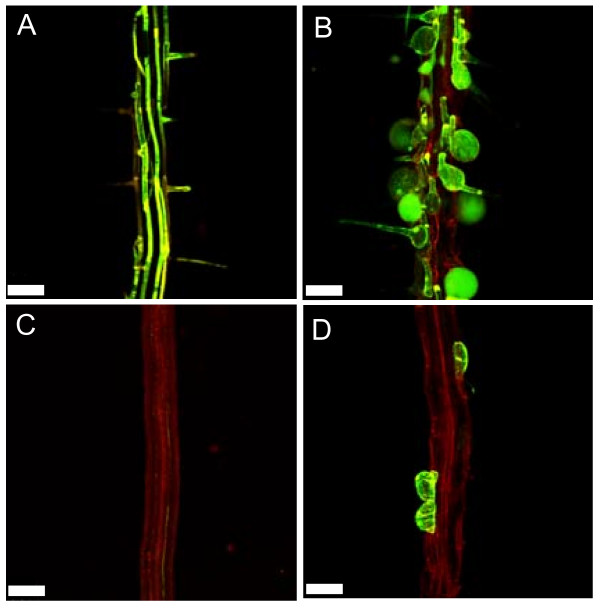
**EXP7 expression which is absent in *rhd6-1 *is restored in a subset of epidermal cells in the *rhd6-1/prc1-1 *double mutant**. The p*EXP7*::*GFP *trichoblast specific marker was introduced into wild-type, *prc1-1*, *rhd6-1 *and *prc1-1/rhd6-1*. Seedlings were grown for 5 days on MS agar and the GFP expression visualized using a Leica fluorescence microscope with a GFP filter. Wild-type (A); *prc1-1 *(B); *rhd6-1 *(C); *prc1-1/rhd6-1 *(D). Bar = 100 μm.

The formation of the root hair bulge is thought to involve the cell wall-loosening activity of expansins and coincides with the expression of the *EXP7 *gene in wild-type roots. EXP7 expression is absent in the *rhd6-1 *mutant [10]. We therefore asked whether the *prc1-1 *mutation would be able to restore *EXP7 *gene expression in the *rhd6-1 *mutant background. The *pEXP7::GFP *marker was crossed into the single *prc1-1 *and *rhd6-1 *mutants as well as the *prc1-1/rhd6-1 *double mutant and its expression examined. Consistent with previous findings [10], *pEXP7::GFP *expression was absent in the *rhd6-1 *mutant (Fig. 6C). However, in agreement with our findings that *prc1-1 *partially rescued root hair formation and induced epidermal bulging in *rhd6-1 *(Fig. 5D), we observed that *pEXP7::GFP *expression was partially restored in the *prc1-1/rhd6-1 *double mutant, where it was specifically observed in those individual epidermal cells which had either formed a root hair and/or exhibited bulging (Fig. 6D). In the *prc1-1 *mutant p*EXP7*::GFP expression was observed in epidermal cells which had either formed bulges or produced a root hair structure (Fig. 6B). These findings link the formation of epidermal bulges with the induction of *EXP7 *expression.

### Is the restoration of hair formation in *rhd6-1 *mediated by *prc1-1 *acting via an ethylene dependent pathway?

**Figure 7:**
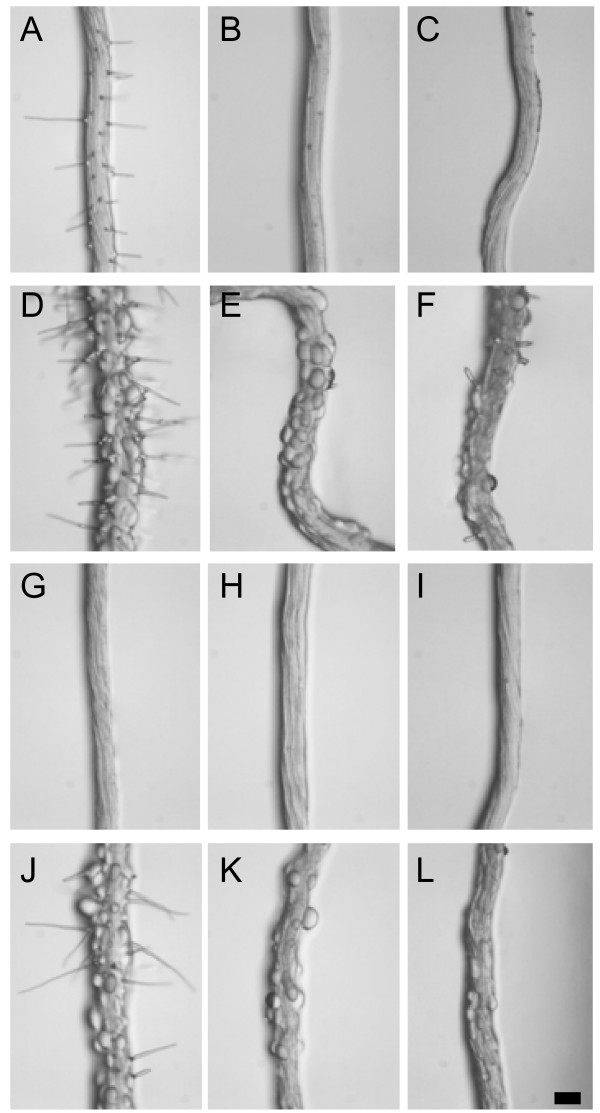
**Is restoration of root hairs in *rhd6-1 *by *prc1-1 *acting via an ethylene dependent pathway? **Root hair development was studied in seedlings grown for 7 days on MS agar containing either no additions (A, D, G, J), 10 μM AgNO_3 _to block the ethylene receptor (B, E, H, K), or 2 μM AVG to block ethylene synthesis (C, F, I, L) Col wild-type (A, B, C), *prc1-1 *(D, E, F), *rhd6-1 *(G, H, I) and *prc1-1/rhd6-1 *(J, K, L). Bar = 100 μm.

The formation of root hairs by wild-type seedlings can be inhibited either by blocking the ethylene receptor using silver ions [25] or via inhibition of ethylene synthesis using aminoethoxyvinyl-Glycine (AVG) [6,26,33]. Conversely, elevated ethylene levels can stimulate root hair formation in wild-type and can result in the restoration of hair formation in *rhd6 *[6]. This raised the question of whether the partial restoration of hair formation in *rhd6-1 *by *prc1-1 *acts via an ethylene dependent pathway. To test this wild-type, *prc1-1*, *rhd6-1 *and *prc1-1/rhd6-1 *seedlings were grown in the presence of 10 μM AgNO_3 _or 2 μM AVG. Treatment with either Ag^+ ^or AVG was equally effective in blocking the formation of hairs by the wild-type and all mutant combinations tested (Fig. 7). Interestingly, although Ag^+ ^and AVG treatment almost completely blocked hair formation by *prc1-1*, there was still a significant degree of epidermal cell bulging although this was reduced compared to roots grown in the absence of inhibitors (Fig. 7D, E, F). It has also been shown that increasing ethylene formation in *prc1 *using 5 μM 1-aminocyclopropoane-1-carboxylic acid (ACC) does not abolish the root epidermal bulging phenotype [27]. Thus the epidermal bulging phenotype of *prc1-1 *is at least partially independent of ethylene whereas the restoration of hair formation in *rhd6-1 *by *prc1-1 *is ethylene dependent (Fig. 7K, L).

### Reduced root elongation does not restore root hairs in *rhd6-1*

**Figure 8:**
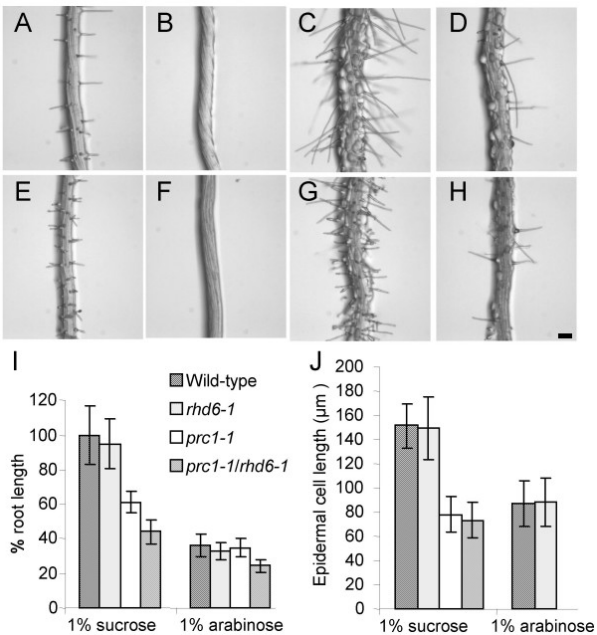
**Reduced root elongation and epidermal cell length does not induce root hair formation in *rhd6-1***. Wild-type (A, E), *rhd6-1 *(B, F), *prc1-1 *(C, G) and *prc1-1/rhd6-1 *(D, H) were grown for 8 days on MS agar containing either 1% sucrose (A-D) or 1% arabinose (E-H). (I) Primary root lengths were measured and expressed as a percentage of the wild-type growth on 1% sucrose. (J) Lengths of mature root epidermal cells of wild-type, *rhd6-1*, *prc1-1 *and *prc1-1/rhd6-1 *grown on 1% sucrose or 1% arabinose (n = 30). Error bars show s.d. Bar = 100 μm.

The *prc1-1 *mutant background results in reduced primary root elongation in *rhd6-1 *coincident with the restoration of hair formation. This raises the possibility that simply reducing the elongation of trichoblast cells is sufficient to induce hair formation in *rhd6-1*. To test this, *rhd6-1 *roots were grown on MS agar lacking sucrose but with the addition of 1 % arabinose which causes reduced primary root elongation [28]. The effect of 1 % arabinose on inhibition of primary root elongation was similar for wild-type and *rhd6-1 *(Fig. 8I). Measurements of the epidermal cell lengths showed that arabinose treatment resulted in a 40 % reduction which is similar to the difference between wild-type and *prc1-1 *or *prc1-1/rhd6-1 *roots grown on 1 % sucrose (Fig. 2B). There was no evidence of root hair formation by *rhd6-1 *grown in the presence of 1 % arabinose (Fig. 8F) in contrast, to the wild-type, *prc1-1 *and the *prc1-1/rhd6-1 *double mutant (Fig. 8E, G, H). Similar results were obtained using 0.3 % xylose, 4 % sorbitol, 3.5 % myo-inositol or 3 % mannitol as the carbon source (data not shown) which resulted in a reduction in *rhd6-1 *root elongation of between 27 % and 60 %. Thus reduced epidermal cell elongation is not a significant contributing factor in the restoration of hair formation in *rhd6-1 *by *prc1-1*.

## Discussion

Within this study we sought to address the role of cell wall strength and loosening in the control of the early steps of hair formation by utilizing the *prc1-1 *mutant in the *CesA6 *cellulose synthesis gene. Our analyses revealed that the radial swelling of trichoblast cells in *prc1-1 *roots is linked to the early steps in hair formation and that the swelling is greatly reduced in the root hairless *rhd6-1 *mutant background. The radial swelling of *prc1-1 *trichoblasts is reminiscent of similar phenotypes exhibited by the *rsw10 *(ribose-5-phosphate isomerase) [17] and *root epidermal bulger 1 *(*reb1/rhd1*) [18] mutants. The *rsw10 *mutant, like *prc1-1*, shows bulging or ballooning of trichoblast cells and has reduced cell wall cellulose content [17]. The *RSW10 *gene is expressed in the distal elongation zone root and within epidermal, cortex and vascular cells of the more mature root tissues. This indicates that the cell wall alteration caused by the *rsw10 *is not limited to the trichoblasts and that bulging of trichoblasts may be linked to root hair initiation and/or outgrowth as shown with *prc1-1 *in this study.

The expression pattern of *PRC1 *as indicated by the p*PRC1*::GUS reporter includes both trichoblast and atrichoblast cell files as well as the underlying cell layers (Fig. 4). However, the possibility that the activity of the *PRC1 *promoter region used does not reflect the true expression pattern of the endogenous gene cannot be ruled out. Despite this, there is no evidence of a preferential trichoblast expression of *PRC1*, strongly suggesting that this cannot by itself explain the predominance of epidermal cell bulging in the hair forming cells of *prc1*. The primary cell wall cellulose biosynthesis complex is believed to consist of 3 different CesA subunits; namely CesA1, CesA3 and CesA6-related [13,16,21,29-32]. The possibility that functionality of the cellulose biosynthesis complex differs in the trichoblast verses atrichoblast files due to the expression pattern or activity of one of the other CesA proteins or other important factors cannot be ruled out. However, alteration of the cellulose composition of the cell wall is not the only factor controlling trichoblast expansion properties. Analysis of the *reb1 *mutant has indicated that AGPs are involved in anisotropic cell expansion [19]. Thus a number of different factors contribute to control the site of expansion of trichoblast cells.

### Mislocalisation of Rop in *prc1-1 *is unlikely to account for the bulges formed by trichoblasts

Root hairs in wild-type Arabidopsis are invariably localised close to the basal ends of trichoblast cells [6,7,23] implying that there are developmental mechanisms to control this positioning. Recent work has shown that auxin provides positional information for hair polarity and that this is mediated at least in part via *AUX1/EIN2/GNOM *acting upstream of the recruitment of Rop GTPases to the hair initiation site [24]. Despite this, relatively little is currently understood about the modifications that take place in the cell wall and where these modifications occur to promote hair bulge formation and its subsequent outgrowth.

The bulging seen in *prc1-1 *trichoblasts occurs over the whole of the outer face of the cell which contrasts with the normal localised bulging associated with the hair initiation site seen in the wild-type. However it is unlikely that the *prc1-1 *bulges are abnormal hair-like structures since normal appearing hairs are often found to develop from the bulged surface. This is further supported by the fact that some limited bulging is found in atrichoblast cells which do not undergo the steps leading to hair initiation. Although Rop is found to be mislocalised in some *prc1-1 *trichoblasts it still remains in a discrete patch even though this is more diffuse than in wild-type cells (Fig. 3E, F, G). Rop mislocalisation is therefore unlikely to directly cause the uncontrolled bulging over the whole of the outer face of some *prc1-1 *trichoblasts. Nevertheless, constitutive expression of the activated GTP-bound form of Rop2 and Rop4 results in bulging of epidermal cells reminiscent of the *prc1-1 *phenotype [4,5]. In this case, this may be as a result of Rop marking the whole of the cell wall for loosening rather than a discrete basally localised patch. Thus, CA-Rop expression in wild-type may lead to ectopic cell wall loosening which mimics the *prc1-1 *phenotype. The reason for mislocalisation of Rop in some but not all *prc1-1 *trichoblasts and the more diffuse distribution (Fig. 3) is unclear but suggests that cell wall structure or composition influences the localization of Rop proteins to a certain extent.

### Hair formation in *rhd6-1 *can be partially rescued by *prc1-1*

Although RHD6 is clearly an important factor for normal hair formation it is evident that its requirement can be bypassed by application of auxin or ethylene to the growth medium [6]. The *RHD6 *gene has been cloned and shown to encode a basic-helix-loop-helix transcription factor [33]. RHD6 was found to accumulate in the nuclei of trichoblast cells within the meristem and elongation zone but disappeared before emergence of the root hair. This confirms that RHD6 acts at an early stage of root hair initiation and prior to the formation of the bulge. Interestingly the closely related gene *RHD6-LIKE1 *(*AtRSL1*) also displays a trichoblast specific pattern similar to *RHD6*. By making the *rhd6/rsl1 *double mutant it was found that the effect on hair development was synergistic indicating that the two transcription factors function together in the regulation of hair initiation. Results presented in this paper show that the *prc1-1 *mutation is able to partially restore the formation of root hairs in the *rhd6-1 *mutant (Fig. 2C, 5D, E, F). It is possible that this partial rescue of the *rhd6-1 *root hair defect may depend on the presence of a functional RSL1. Although this has not been tested, it is apparent that the presence of RSL1 in an *rhd6-1 *mutant background is insufficient to promote the formation of root hairs unless the growth conditions are altered such as growing on cellophane [33] or with addition of auxin or ethylene [34]. While the mechanism for the *prc1-1 *mediated partial restoration of root hairs in *rhd6-1 *remains unclear, clues are provided by the fact that hair formation in the double mutant is accompanied by expression of *EXP7 *which is normally absent in an *rhd6-1 *background. It has previously been shown that *EXP7 *and *EXP18 *expression in *rhd6-1 *is restored by hormonal and environmental treatments which induce root hair formation [10]. These findings are consistent with the idea that *EXP7 *may act to loosen the cell wall and that this promotes hair formation. This is further supported by the finding that the bulged regions of trichoblasts are enriched with expansin [11]. While results in this study also support the conclusion that *RHD6 *does not directly control transcription of *EXP7*, the functional role of *EXP7 *still needs clarification, since loss-of-function mutants do not display a phenotype [10].

### Restoration of root hairs in *prc1-1/rhd6-1 *occurs via an ethylene-dependent pathway

Root hair formation is dependent on ethylene production and perception and can be blocked using synthesis inhibitors such as AVG or silver as an inhibitor of the ethylene receptor. Previous studies have shown that *prc1 *seedlings grown on ACC exhibited a triple response and therefore that the mutant was sensitive to ethylene. This also indicates that *prc1-1 *is unlikely to overproduce ethylene since it does not exhibit the triple response in the absence of exogenous ethylene. Additionally, the double mutant between *prc1-1 *and the ethylene insensitive *ein2-1 *did not abolish the growth defects of the *prc1-1 *mutant [22]. This shows that the *prc1-1 *growth defects are likely to be independent of ethylene. Results in this study show that while AVG and Ag^+ ^can effectively block the formation of root hairs, the *prc1-1 *epidermal cells still exhibit a bulging phenotype (Fig. 7E, F). It has also been found that ethylene is unable to rescue the bulging phenotype in *prc1-1 *[27]. Thus the bulging phenotype is at least partially independent of ethylene action. The possibility that the *prc1-1 *mutation results in overproduction of ethylene within root epidermal cells cannot be discounted at present. However it is not possible to measure ethylene production specifically in root epidermal cells to address this question.

### A model for cell wall loosening in root hair initiation

Our results indicate that in wild-type trichoblasts targeting of Rop GTPases to a discrete basal location marks that region for localised cell wall loosening. This poses the question of how cell wall loosening is restricted to the Rop site. One mechanism may be via a localised increase in apoplastic pH via activation of potassium channels [12] thereby inducing expansin activity. In the *prc1-1 *mutant this process is affected in several ways. Firstly, the modification of the cell wall structure can cause an apical or basal shift and some smearing of the Rop localisation although as yet it is not understood why this occurs. It is proposed that the normal increase in turgor pressure occurs as in wild-type but since the whole of the *prc1-1 *cell wall may be weak the entire outer face bulges out instead of just the position where Rop is localised. Interestingly root hairs can still be formed from the bulges suggesting that either Rop or another unknown site-specific signal is still able to direct the normal post bulge steps in hair formation.

It is feasible that *RHD6 *is normally required to direct the cellular machinery causing cell wall loosening and so in an *rhd6 *mutant this does not occur. By placing *rhd6-1 *in a *prc1-1 *mutant background the requirement for cell wall loosening is fulfilled independently of RHD6 and hair initiation can proceed. It is interesting that in *prc1-1/rhd6-1 *roots, *EXP7 *expression is found specifically in cells which are bulged or form a root hair. This indicates that *EXP7 *is an important factor in root hair formation. One intriguing possibility is that EXP7 is induced by radial expansion of trichoblast cells. In wild-type this would require initial loosening of the cell wall possibly via acidification and XET activity. In *rhd6-1 *the cell wall loosening may be blocked and therefore there is no induction of *EXP7*. However in *prc1-1/rhd6-1 *roots the cell wall is already sufficiently weakened in a subset of cells to allow the turgor pressure to cause radial expansion, in turn causing a feedback induction of *EXP7 *expression. This would cause further loosening of the wall and allow root hair formation to proceed.

## Conclusion

The *prc1-1 *mutant has provided useful information showing that the cell wall structure influences the morphogenesis of root hairs and also provided information about the role of *RHD6 *in hair formation. By affecting the cell wall structure and composition in the *prc1 *mutant, the requirement for a functional RHD6 in root hair formation can be partially bypassed. However, further work is required to elucidate the complex interplay between the different genetic and physiological factors which combine to determine the site of hair formation and its subsequent biogenesis.

## Methods

### Growth of Arabidopsis

Wild-type (Columbia 0 ecotype) and mutant seed was surface sterilized [35] and sown *in vitro *on Murashige and Skoog (MS) agar (4.3 g MS salts (Duchefa, Haarlem, Netherlands); 1 % sucrose, pH 5.8 with 0.5 M KOH; 1 % plant agar; Duchefa, Haarlem, Netherlands). Filter sterilized silver nitrate (Fisher Scientific UK Ltd, Loughborough UK) or AVG (Sigma Aldrich, Steinheim, Germany) was added to media after autoclaving where appropriate. The seed was chilled at 4°C for 48 hours prior to germination under a 16 hour light period, 120 μmol m^-2- ^s^-1 ^light intensity (Philips TL-D 36W/840 bulbs) at 23°C unless otherwise stated. Soil grown plants were maintained in controlled growth rooms with a light intensity of 120 μmol m^-2- ^s^-1 ^at 23°C under a 16 hour light period.

### Analysis of root tissues

Root hair numbers were counted for wild-type, *prc1-1*, *rhd6-1*, and *prc1-1/rhd6-1 *seedlings that had been grown for 7 days on vertically oriented MS agar plates. The roots were mounted in a solution of chloral hydrate:glycerol:H_2_O 8:3:1 and left overnight at 20°C to clear. The roots were viewed using an Axioplan 2 microscope (Zeiss, Oberkochen, Germany) and the numbers of root hairs counted in a single trichoblast cell file within a 1 mm region adjacent to the differentiation zone for 100 independent roots. Root trichoblast cell lengths and the proportion of trichoblast cells forming a root hair were determined in mature root tissues that had fully elongated for wild-type, *prc1-1*, *rhd6-1 *and *prc1-1/rhd6-1*. Seedlings were grown in 3 separate biological replicates and 20 cells measured from each of 10 separate roots to give a total of 600 cells for each genotype. The occurrence and positioning of bulged epidermal cells was determined in radial sections of resin embedded roots. Cells were scored as to whether they were bulged or unbulged and whether they were in trichoblast or atrichoblast positions (n = 30 independent roots per genotype).

pEXP7::GFP expression in root tissues was visualised using a Leica MZIII fluorescence microscope with a GFP filter and images captured using a Leica DC100 camera. Root tissues were prepared and sectioned as previously described [36]. Tissue sections were stained with ruthenium red (0.05 %) and viewed using an Axioplan 2 microscope and images captured using an Axiocam camera.

### Measurement of root hair position

Seedlings were mounted in a solution of chloral hydrate:glycerol:H_2_O 8:3:1 and the relative root hair position determined by dividing the trichoblast cell length by the distance from the basal cell wall of the trichoblast to the basal wall of the root hair. Roots were viewed using an Axioplan 2 microscope employing Axiovision 3.1 software for image capture and measurements. For each genotype, at least 150 cells were measured from 3 independent experiments each using 10 roots and 5 trichoblasts per root. Data was analysed for significance employing the Fisher Exact test and Mann-Whitney tests as previously described [7,24].

### Immunolocalisation of Rop GTPase

Fixation and preparation of root tips for immunolocalisation of the Rop GTPase was performed based on the protocol of Grebe and coworkers [37] with the modifications introduced by Fischer and coworkers [24]. Blocking was performed using 5% donkey serum (Jackson ImmunoResearch, West Grove, PA). The primary rabbit anti-Rop antibody was used at a 1:50 dilution and the secondary donkey anti-rabbit FITC-coupled antibody (Jackson ImmunoResearch, West Grove, PA) was diluted 1:250. Prior to mounting in Citifluor AF1 (Citifluor, London UK) the samples were stained with DAPI (1 μg/ml). CLSM was performed as described [24] employing a Leica TCS SP2 AOBS scanning system mounted on an inverted Leica DM IRE2 microscope and employing Leica TCS software. Excitation was performed using a 405 nm diode laser (DAPI) and 488 nm argon laser (FITC). Emission wavelengths were detected between 410 and 500 nm for DAPI and 500 and 550 nm for FITC. Pictures from sequential scans were overlayed using Adobe Photoshop 7.0 and assembled in Adobe Illustrator 9.0.

## List of abbreviations

XET: Xyloglucan endotransglycosidase; EXP: expansin; AVG: aminoethoxyvinyl glycine; ACC: 1-aminocyclopropane-1-carboxylic acid; MS: Murashige and Skoog; AGP: arabinogalactan protein.

## Availability & requirements

Fisher Exact test: 

Mann-Whitney rank sum test: 

## Authors' contributions

SKS carried out mutant analysis and reporter studies, UF performed the Rop localisation experiments and MS generated and characterized the Rop antibody. MG was involved in experimental design and analysis of results. AM conceived the study, and participated in its design and coordination and wrote the manuscript. All authors read and approved the final manuscript
